# Change in systemic steroid use and surgery rate in patients with inflammatory bowel disease: a Japanese real-world database analysis

**DOI:** 10.1007/s00535-024-02086-y

**Published:** 2024-03-16

**Authors:** Daisuke Hirayama, Shinichiro Hyodo, Kazuo Morita, Hiroshi Nakase

**Affiliations:** 1 Department of Gastroenterology and Hepatology, Sapporo Medical University School of Medicine, S1 W17, Chuo-Ku, Sapporo-Shi, Hokkaido, 060-8556 Japan; 2 AbbVie GK, 3-1-21 Shibaura, Minato-Ku, Tokyo, 108-0023 Japan

**Keywords:** Biologic agents, Inflammatory bowel diseases, Steroids

## Abstract

**Background:**

Corticosteroids are recommended only for induction of remission in inflammatory bowel disease (IBD), including ulcerative colitis (UC) and Crohn’s disease (CD). This study aimed to evaluate the change in pharmacologic treatment use, particularly systemic corticosteroids, over approximately 30 years, and the impact of biologics on IBD treatment since their appearance in the 2000s.

**Methods:**

This retrospective study conducted in Japan used data from the Phoenix cohort database (January 1990 to March 2021). Patients with disease onset at age ≥ 10 years who received treatment for UC or CD between January 1990 and March 2021 were included. Outcome measures were change in IBD treatments used, total cumulative corticosteroid doses, initial corticosteroid dose, duration of corticosteroid treatment, and surgery rate.

**Results:**

A total of 1066 and 579 patients with UC and CD, respectively, were included. In UC, the rate of corticosteroid use as initial treatment was relatively stable regardless of the year of disease onset; however, in CD, its rate decreased in patients who had disease onset after 2006 (before 2006: 14.3–27.8% vs. after 2006: 6.6–10.5%). Compared with patients with disease onset before biologics became available, cumulative corticosteroid doses in both UC and CD, and the surgery rate in CD only, were lower in those with disease onset after biologics became available.

**Conclusions:**

Since biologics became available, corticosteroid use appears to have decreased, with more appropriate use. Furthermore, use of biologics may reduce surgery rates, particularly in patients with CD. UMIN Clinical Trials Registry; UMIN000035384.

**Supplementary Information:**

The online version contains supplementary material available at 10.1007/s00535-024-02086-y.

## Introduction

Inflammatory bowel disease (IBD), which mainly includes ulcerative colitis (UC) and Crohn’s disease (CD), is a chronic, relapsing inflammatory disorder of the gastrointestinal tract [1]. UC is characterized by continuous mucosal inflammation of the colon and the rectum [2], whereas CD is associated with discontinuous transmural inflammation with ulcerous lesions in any part of the gastrointestinal tract [3]. Patients with UC and CD may present with symptoms including abdominal pain, diarrhea, weight loss, and rectal bleeding [4], and both diseases are associated with a poor health-related quality of life [5, 6]. The prevalence of IBD is increasing globally [7], including in Japan [8]. In Japan, more than 219,000 and 70,000 patients were estimated to have UC and CD, respectively, in 2014, with prevalence rates of 172.9 and 55.6 per 100,000 population [8].

Currently, treatment for IBD includes 5-aminosalicylic acid (5-ASA), corticosteroids, immunomodulators (e.g., azathioprine and 6-mercaptopurine), biologics (e.g., infliximab, adalimumab, vedolizumab, and ustekinumab), and Janus kinase inhibitors (e.g., tofacitinib) [9]. In the past, IBD treatment relied on surgery, 5-ASA, corticosteroids, and nutritional therapy [10, 11]. However, the introduction of immunomodulators and biologics has broadened the treatment options and improved treatment outcomes. In Japan, the first immunomodulator approved was azathioprine in 2006. Infliximab was the first biologic approved for moderately to severely active CD in 2002, with an indication added for CD maintenance therapy in 2007, and then for UC induction and maintenance therapy in 2010. This was followed by adalimumab for CD in 2010 and for UC in 2013. Treatments are selected depending on factors that include the patient’s general condition, disease severity, and disease status, with the aim of inducing and maintaining remission, and decreasing hospitalization and surgery [9, 12]. In the current international (including Japanese) IBD guidelines, corticosteroids are only recommended for inducing remission [9]. This is because corticosteroids lack efficacy in maintaining remission, and their long-term use should be avoided due to the risk of side effects, including infection and osteoporosis [13–15].

Given the advances in IBD treatment, it is important to understand the real-world use of IBD treatments and how treatment patterns have evolved over the years. However, because previous studies in Japan have focused on either UC or CD and have evaluated the changes in treatment since the late 2000s [15–17], information on the real-world use of treatments for both UC and CD in Japan is still limited, particularly related to the impact of the availability of biologics on treatment patterns, including surgery rates. Therefore, further real-world study is required to improve understanding of the IBD treatments used in Japan.

This retrospective cohort study conducted in Hokkaido, Japan (UMIN Clinical Trials Registry; UMIN000035384) aimed to gain further insights into the changes in pharmacologic treatments (particularly systemic corticosteroids) used for IBD over approximately 30 years from 1990 to 2021, and how the availability of biologic treatments has affected surgery rates in Japan.

## Material and methods

### Study design

This was a retrospective study conducted in the Hokkaido region of Japan using a database originally developed for the *P*rincipal research in the *H*okkaido *O*rganization *E*mphasizing *N*utritional and therapeutic *I*mprovement to IBD patients’ e*X*pectation (Phoenix) Cohort Study (UMIN Clinical Trials Registry; UMIN000035384), which was initiated in October 2018 and aimed to collect data of patients with IBD and develop new diagnostic criteria and a treatment guideline for IBD. The Phoenix cohort database consisted of data for approximately 2100 patients with IBD who were treated at five core IBD treatment hospitals (Sapporo Medical University Hospital, Hokkaido University Hospital, Asahikawa Medical University Hospital, Sapporo-Kosei General Hospital, and Sapporo Higashi Tokushukai Hospital) between January 1, 1990, and March 31, 2021, which have been used for previous studies of IBD [18, 19]. Patient data were collected using medical records and included clinical findings (e.g., treatment details, dosing status, treatment duration, and outcomes), blood test results, endoscopy findings, pathology, and imaging examination findings. The Phoenix study was approved by the ethics review boards at each study site and was conducted in accordance with the Declaration of Helsinki, Japan’s Ethical Guidelines for Medical and Health Research Involving Human Subjects, and all relevant laws and regulations. Because the current study reused de-identified data from the Phoenix study, ethical review was not required. Informed consent was also not required, and the patients were given the opportunity to “opt out” at the time of participation and at any time during the Phoenix study.

### Study population

Patients who received treatment for either UC or CD between January 1990 and March 2021, and who had an onset of disease at age ≥ 10 years, were identified from the database and were included in the study. Patients with unclassified IBD were excluded from the study.

### Outcome measures

The primary end point was the percentage of patients using each type of IBD treatment (corticosteroids, immunomodulators [thiopurine agents], biologics, and 5-ASA) as an initial treatment, by year of onset of disease. Secondary end points included the following: total cumulative corticosteroid dose from onset of disease for which the corticosteroid was started; change in initial corticosteroid dose over different time periods; change in duration of corticosteroid treatment over different time periods, which was calculated from the start date of corticosteroid treatment to the date of last consultation; and surgery rates after disease onset. Initial treatment was defined as the treatment that was administered until remission. Corticosteroids were defined as systemic corticosteroids (oral or intravenous prednisolone and methylprednisolone); intestinal selective corticosteroids including budesonide and rectal formulations were excluded. Corticosteroid doses were reported as prednisolone equivalent.

### Statistical analysis

Because of the observational nature of this study, no target sample size was set, and all eligible patients from the database were included. Data were summarized for the overall population and the corticosteroid-treated subpopulation for both UC and CD using descriptive statistics. Categorical variables are presented as n (%), and continuous variables are presented as mean (standard deviation) and/or median (minimum, maximum, interquartile range [IQR]). The mean cumulative corticosteroid dose was calculated for subgroups of patients who had a disease onset before and after biologics became available. For patients who had an onset of disease before biologics became available, corticosteroids administered after the date when biologics first became available were excluded from the calculation. Surgery rates by onset of disease before and after the launch of biologics were analyzed in the UC and CD overall populations using the Kaplan–Meier method. “Before biologics” was defined as any time before the year of infliximab approval, i.e., up to and including 2002 for CD, up to and including 2007 for CD maintenance therapy (for analysis of surgery rate only), and up to and including 2010 for UC. “After biologics” was defined as any time after the year of infliximab approval, i.e., starting in 2003 for CD, 2008 for CD maintenance therapy (for surgery rate only), and 2011 for UC. Missing data were not imputed. Statistical analyses were performed using SAS 9.4 (SAS Institute Inc., Cary, NC, USA) and Microsoft Office 2010 or later (Microsoft Corporation, Redmond, WA, USA).

## Results

### Demographic and baseline clinical characteristics

**Fig. 1:**
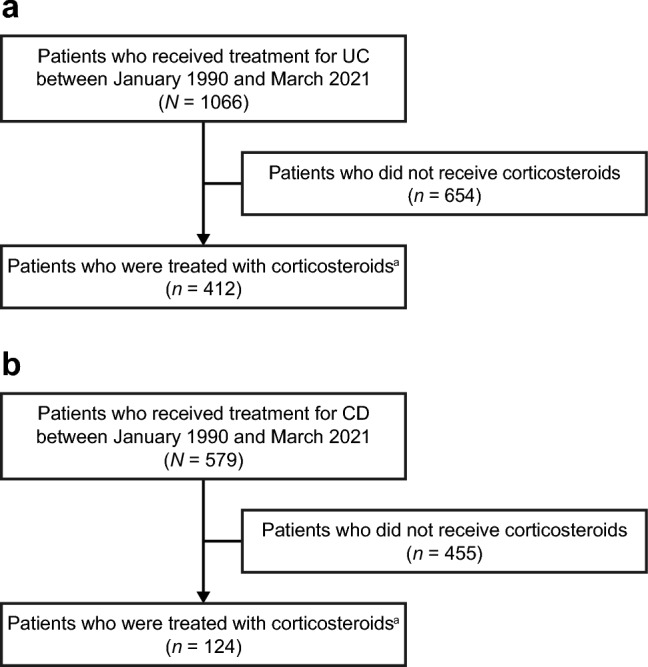
Patient disposition for **a** UC and **b** CD. ^a^ Patients who started corticosteroid treatment before the onset of disease were excluded. *CD* Crohn’s disease, *UC* ulcerative colitis

The Phoenix cohort database included 1066 and 579 patients who were treated for UC and CD, respectively, between January 1990 and March 2021 (Fig. 1), from Sapporo Medical University Hospital (UC: *n* = 187, CD: *n* = 90), Hokkaido University Hospital (UC: *n* = 80), Asahikawa Medical University Hospital (UC: *n* = 273, CD: *n* = 205), Sapporo-Kosei General Hospital (UC: *n* = 257, CD: *n* = 146), and Sapporo Higashi Tokushukai Hospital (UC: *n* = 269, CD: *n* = 138). Of these, 412 patients with UC and 124 patients with CD were treated with systemic corticosteroids at least once. The median (IQR) age of onset of disease was 34.0 (24.0–47.0) years in patients with UC and 22.0 (18.0–29.0) years in patients with CD (Table 1). More patients with CD were male than female. The most common disease types were pancolitis (47.7%) for UC and ileocolonic type (65.3%) for CD. Most patients had no extraintestinal manifestations for both UC and CD. Furthermore, baseline characteristics were generally similar across patients with different years of disease onset in the overall population (Supplementary Table 1 and 2) and in the corticosteroid-treated subpopulation for both UC and CD (Supplementary Table 3, 4, 5).

**Table 1:** Patient baseline demographics and clinical characteristics for the overall population

Characteristics	UC (*n* = 1066)	CD (*n* = 579)
Sex, *n* (%)		
Male	514 (48.2)	427 (73.7)
Female	549 (51.5)	151 (26.1)
Unknown	3 (0.3)	1 (0.2)
Duration of disease		
* n* (%)	1055 (99.0)	577 (99.7)
Median (IQR), years	6.0 (3.0–12.0)	7.0 (4.0–17.0)
Age at onset of disease		
* n* (%)	1060 (99.4)	578 (99.8)
Median (IQR), years	34.0 (24.0–47.0)	22.0 (18.0–29.0)
Height		
* n* (%)	820 (76.9)	456 (78.8)
Median (IQR), cm	163.0 (157.0–170.0)	168.0 (160.0–173.0)
Weight		
* n* (%)	807 (75.7)	433 (74.8)
Median (IQR), kg	57.0 (49.2–67.0)	55.0 (49.0–63.0)
Drinking history, *n* (%)		
Yes	339 (31.8)	128 (22.1)
No	537 (50.4)	344 (59.4)
Unknown	190 (17.8)	107 (18.5)
Smoking history, *n* (%)		
Current smoker	119 (11.2)	89 (15.4)
Past smoker	111 (10.4)	24 (4.1)
Non-smoker	655 (61.4)	361 (62.3)
Unknown	181 (17.0)	105 (18.1)
Disease type, *n* (%)		
Proctitis	253 (23.7)	–
Left-sided colitis	234 (22.0)	–
Pancolitis	509 (47.7)	–
Regional colitis	4 (0.4)	–
Ileal	–	125 (21.6)
Colonic	–	68 (11.7)
Ileocolonic	–	378 (65.3)
Isolated upper disease	–	1 (0.2)
Unknown	66 (6.2)	7 (1.2)
Extraintestinal manifestations, *n* (%)		
Yes	73 (6.8)	74 (12.8)
No	844 (79.2)	448 (77.4)
Unknown	149 (14.0)	57 (9.8)
Anal lesions		
Yes	–	259 (44.7)
No	–	249 (43.0)
Unknown	–	71 (12.3)

*CD* Crohn’s disease, *IQR* interquartile range, *UC* ulcerative colitis

### Drugs used as initial treatment for UC, by year of disease onset

**Fig. 2:**
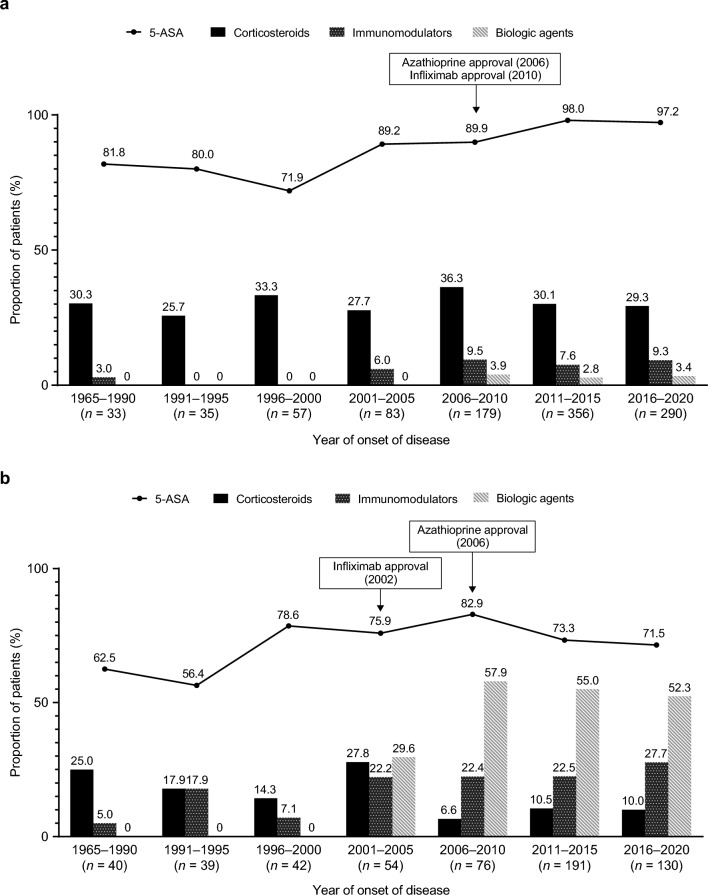
Percentage of patients using each type of drug as initial treatment, by 5-year period of onset of disease in overall population with **a** UC and **b** CD. *5-ASA* 5-aminosalicylic acid, *CD* Crohn’s disease, *UC* ulcerative colitis

Among patients with UC, 5-ASA was the most common drug prescribed as an initial treatment, with 71.9–98.0% of patients receiving 5-ASA regardless of the year of disease onset (Fig. 2A). The proportion of patients receiving corticosteroids as initial treatment ranged between 25.7% and 36.3% across patients with different years of disease onset. Immunomodulators were prescribed in ≤ 3% of patients who had an onset of disease before 2000 but this ranged from 6.0% to 9.5% in those who had an onset of disease after 2001. The proportion of patients prescribed biologics ranged from 2.8% to 3.9% in those with onset of disease between 2006 and 2020, reflecting the approval of infliximab for UC in 2010.

### Drugs used as initial treatment for CD, by year of disease onset

Among patients with CD, 5-ASA was prescribed as an initial treatment to 56.4–82.9% of patients regardless of the year of disease onset; however, the proportion was slightly higher in those with a disease onset after 1996 compared with those with a disease onset before 1996 (Fig. 2B). The proportion of patients with corticosteroids as initial treatment ranged from 6.6% to 27.8%, but corticosteroid use was generally lower in patients who had an onset of disease after 2006 than in those who had an onset of disease between 1965 and 2005. The proportion of patients prescribed immunomodulators ranged from 5.0% to 27.7%, with higher use in patients who had an onset of disease after 2001 than in those who had an onset of disease between 1965 and 2000. After the approval of infliximab for CD in 2002, biologic use increased to 29.6% in those with an onset of disease between 2001 and 2005, and to > 50% in those with an onset of disease between 2006 and 2020.

### Cumulative corticosteroid dose use in UC and CD

Among patients with UC, the cumulative corticosteroid dose was numerically lower in those who had an onset of disease after biologics became available compared with those who had an onset of disease before biologics became available (Table 2). Similarly, among patients with CD, mean cumulative corticosteroid dose in those who had an onset of disease before biologics became available was numerically higher than in those who had an onset of disease after biologics became available (Table 2).

**Table 2:** Cumulative corticosteroid dose before and after biologic agents became available

	Disease onset before biologics became available	Disease onset after biologics became available
UC, *n*	87	175
Cumulative corticosteroid dose, mg		
Mean (SD)	5710.3 (7667.3)	2484.9 (1889.2)
Median (min, max)	3000.0 (770.0, 40,000.0)	1855.0 (20.0, 12,398.5)
Duration of disease, days		
Mean (SD)	6618.5 (3130.0)	1650.3 (908.6)
Median (min, max)	5444.0 (2061.0, 19,775.0)	1537.0 (70.0, 3450.0)
CD, *n*	12	49
Cumulative corticosteroid dose, mg		
Mean (SD)	7429.5 (9373.7)	2013.3 (1456.4)
Median (min, max)	3071.3 (300.0, 31,990.0)	1732.5 (150.0, 6606.0)
Duration of disease, days		
Mean (SD)	10,574.3 (1921.0)	2753.0 (1659.0)
Median (min, max)	10,629.5 (7140.0, 13,917.0)	2537.0 (159.0, 6221.0)

“Before biologics” was any time before the year of infliximab approval, i.e., up to and including 2002 for CD, and up to and including 2010 for UC. “After biologics” was any time after the year of infliximab approval, i.e., starting in 2003 for CD, and 2011 for UC. For patients who had an onset of disease before biologics became available, corticosteroids administered after the date when biologics first became available were excluded from the calculation. Duration of disease data are for reference only; corticosteroids are recommended for short-term use only to induce remission, and therefore patients may not have been treated with corticosteroids continuously for the whole duration of the disease

*CD* Crohn’s disease, *max* maximum, *min* minimum, *SD* standard deviation, *UC* ulcerative colitis

### Initial corticosteroid dose and duration of treatment in UC and CD

**Fig. 3:**
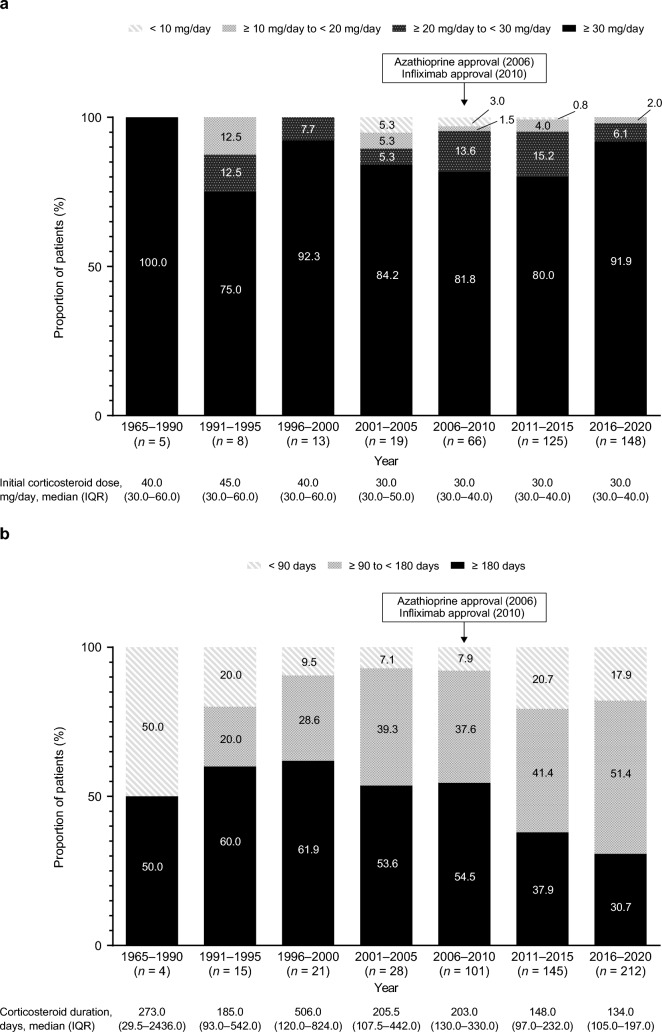
**a** Initial corticosteroid dose and **b** duration of corticosteroid treatment in patients with UC. The proportion of patients in each period is calculated by including those who started corticosteroid treatment in the respective period. *IQR* interquartile range, *UC* ulcerative colitis

**Fig. 4:**
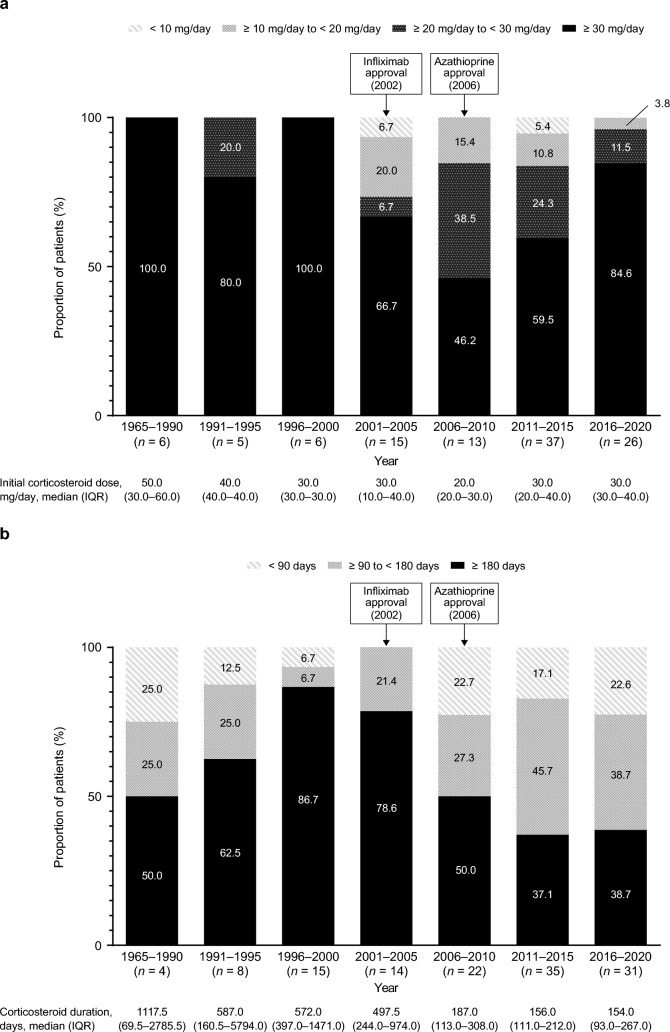
**a** Initial corticosteroid dose and **b** duration of corticosteroid treatment in patients with CD. The proportion of patients in each period is calculated by including those who started corticosteroid treatment in the respective period. *CD* Crohn’s disease, *IQR* interquartile range

Among the corticosteroid-treated patients with UC, most patients (≥ 75%) received an initial corticosteroid dose of ≥ 30 mg/day, with approximately < 5% of patients receiving an initial dose of < 10 mg/day, across all time periods (Fig. 3A). The proportion of patients receiving corticosteroids for ≥ 180 days decreased between 2001 and 2020; conversely, the proportion receiving corticosteroids for < 90 days generally increased during that period (Fig. 3B). Among the corticosteroid-treated patients with CD, the proportion of patients receiving an initial corticosteroid dose of ≥ 30 mg/day increased between 2006 and 2020, with > 45% of patients receiving ≥ 30 mg/day across the three 5-year periods (Fig. 4A). Over 75% of patients received corticosteroids for ≥ 180 days in 1996–2005; however, this decreased to ≤ 50% between 2006 and 2020 (Fig. 4B). The proportion of patients receiving corticosteroids for < 90 days decreased between 1965 and 2005 and generally increased after 2006; the proportion receiving corticosteroids for 90–179 days also decreased from 1965 to 2000 but increased from 2001 to 2020.

### Surgery rate in UC and CD

**Fig. 5:**
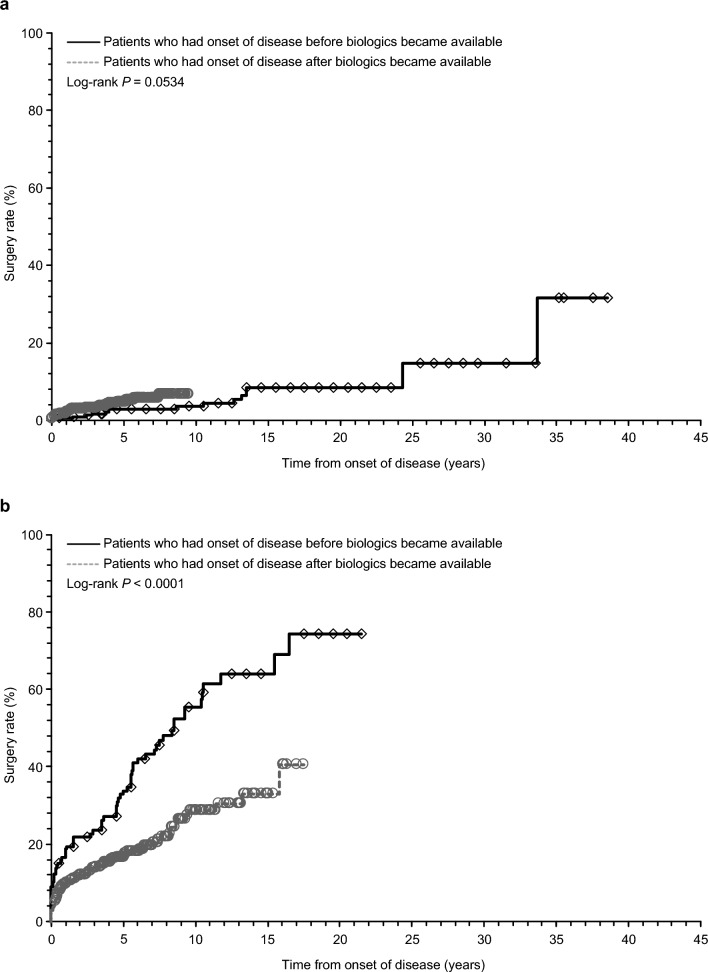
Kaplan–Meier curve of surgery rate in corticosteroid-treated patients with **a** UC and **b** CD. “Before biologics” was any time before the year of infliximab approval, i.e., up to and including 2002 for CD, and up to and including 2010 for UC. “After biologics” was any time after the year of infliximab approval, i.e., starting in 2003 for CD, and 2011 for UC. *CD* Crohn’s disease, *UC* ulcerative colitis

In the overall UC population, the surgery rate was numerically higher in patients who had an onset of disease after biologics became available compared with those who had an onset of disease before biologics became available (Fig. 5A). However, the surgery rate was low (< 20%) in both patient groups at all time points up to 9 years from the onset of disease. In the overall CD population, the surgery rate was substantially lower in patients who had an onset of disease after biologics became available in 2002 (i.e., when infliximab was first approved for CD) compared with those who had an onset of disease before biologics became available in 2002, at all time points up to 17 years from the onset of disease (Fig. 5B). At this time point, the surgery rate in patients with an onset of disease after biologics became available was almost half that observed in patients who had an onset of disease before biologics became available. Similar results were observed before and after 2007 (i.e., before and after infliximab was approved for CD maintenance therapy; Supplementary Figure). Among patients who had an onset of disease before biologics became available, the surgery rate reached a plateau of 67.2% at 17 years from the onset of disease. Among patients who had an onset of disease after biologics became available, the surgery rate reached a plateau of 27.1% at 10 years from the onset of disease.

## Discussion

Using data from the Phoenix study, we analyzed the real-world treatment patterns, especially the use of systemic corticosteroids, over approximately 30 years in patients with UC or CD in Japan. Corticosteroid use as initial treatment was relatively stable in patients with UC but decreased in patients with CD who had an onset of disease after 2006, compared with those who had an onset between 1965 and 2005. In both UC and CD, cumulative corticosteroid doses were numerically lower in patients with an onset of disease after biologics became available than in those with an onset of disease before biologics became available. In CD, the proportion of patients receiving an initial corticosteroid dose of ≥ 30 mg/day increased from 2006 to 2020, with a decreasing trend in the proportion receiving long-term treatment (≥ 180 days). Patients with CD who had an onset of disease after biologics became available had a substantially lower surgery rate compared with those with an onset of disease before biologics became available. In UC, although less clear, the proportion of patients receiving long-term corticosteroid treatment decreased after 2001; however, no apparent trend in changes to initial corticosteroid dose was observed. Furthermore, in contrast to CD, the surgery rate appeared slightly higher in those with an onset of disease after biologics became available than in those with an earlier onset of disease. These results suggest that the availability of biologics has contributed to the more appropriate use of corticosteroids and reduced surgery rates, especially in patients with CD, although the use of corticosteroids in IBD can be further improved, particularly by using newer treatments that are available.

This study shows that the proportion of patients initially treated with corticosteroids was lower in those with a later disease onset than in those with an earlier disease onset in CD, but not in UC. Although a direct comparison of the results is not possible, a population-based analysis in Canada reported that patients receiving corticosteroids at any time decreased between 1997 and 2017 in both UC and CD [20]. In a previous retrospective database study of Japanese patients with UC, the proportion of patients receiving corticosteroids at any time decreased between 2009 and 2013 [17]. Another database study of Japanese patients with UC showed that corticosteroid use in UC decreased between 2006 and 2016 [15]. The Japanese guidelines for UC and CD were first published in 2006 and 2010, respectively [21]; therefore, no local guidelines were available for those with a disease onset before these years. Currently, corticosteroids are recommended only to induce disease remission [9, 22, 23]. European guidelines recommend 5-ASA and corticosteroids for mild-to-moderate UC, but only corticosteroids for active CD [22, 23]. Japanese guidelines recommend 5-ASA for mild-to-moderate UC (with corticosteroids only for patients who are unresponsive to 5-ASA), and both 5-ASA and corticosteroids for active CD [9]. In relation to these recommendations, UC is often treated using a “step-up” approach, whereas CD, particularly since biologics became available, is treated using a “top-down” approach [24, 25]. Therefore, the publication of the treatment guidelines, together with the differences in treatment approach and the approval of immunomodulators and biologics in Japan in the mid-2000s, may explain why a decreasing trend in corticosteroid use as initial treatment was observed in patients with CD, although no clear trend was observed in patients with UC. However, it is important to note that the results from the current study suggest that approximately one-third of patients with UC were treated with corticosteroids, either alone or in combination with other drugs (e.g., 5-ASA), as initial treatment regardless of the year of disease onset. Furthermore, in this study, the proportion of patients with CD prescribed corticosteroids as initial treatment was lowest in those who had a disease onset after 2006 (< 15%); in fact, the proportion of patients prescribed corticosteroids was lower than those treated with immunomodulators or biologics. Nonetheless, these results indicate that the overall use of corticosteroids has decreased in recent years since the approval of biologics, and that corticosteroids are used more appropriately as remission induction therapy, in line with the current treatment guidelines [9].

The dose and duration of corticosteroids affect the incidence and severity of most corticosteroid-related adverse events [26]. The current Japanese treatment guideline recommends oral prednisolone 30–40 mg/day for UC and 40 mg/day for CD initially, reducing to < 10 mg/day within 3 months [9, 14] to minimize the potential long-term adverse events associated with corticosteroids [13]. In a previous Japanese database study of UC conducted between 2006 and 2016, low initial corticosteroid dose was associated with long-term use (≥ 180 days) of corticosteroids (*P* < 0.001) [15]. This same database study showed that the proportion of patients using an initial corticosteroid dose ≥ 30 mg/day gradually increased between 2006 and 2016. Conversely, in the current study, most patients with UC received an initial corticosteroid dose of ≥ 30 mg/day across all time periods with no apparent trend of changes in initial dose over time between 1965 and 2020. However, the proportion of patients using corticosteroids ≥ 30 mg/day was substantially higher in this study than in the previous database study [15], and the proportion of patients treated for ≥ 180 days generally decreased after 2001. In contrast, in patients with CD, a gradual increase in the proportion of patients receiving an initial corticosteroid dose of ≥ 30 mg, with a gradual decrease in those treated for ≥ 180 days, was observed after 2006. In both UC and CD, the availability of biologics also appeared to have reduced the cumulative corticosteroid doses used. However, it should be noted that the sample size was limited, and the disease duration was longer in patients who had a disease onset before biologics became available. Although corticosteroids are recommended for short-term use (≤ 3 months) only [9], during the remission induction phase, there is a possibility that some patients may have had extraintestinal complications (e.g., pyoderma gangrenosum) that may require treatment with long-term corticosteroids. The results from this study collectively indicate that the corticosteroid doses used are in line with the current guideline for most patients with UC or CD, reducing the proportion of patients being treated for a long duration (≥ 180 days) after the appearance of biologics. However, because there are still patients who are treated for an intermediate duration (90–179 days), it may be important to consider the use of immunomodulators and biologics where appropriate to further reduce long-term corticosteroid use, thus preventing corticosteroid-related complications.

During the course of the disease, surgery will be required in 35% of patients with UC and up to 70% of patients with CD [27]. However, surgery is associated with postoperative complications, such as suture failure and intestinal obstruction in UC and short bowel syndrome in CD [9]. Therefore, current treatment guidelines recommend surgery in patients with severe disease or in those with cancer or dysplasia [9]. Results from the phase 3, randomized, placebo-controlled CHARM trial [28] of adalimumab also showed that the major surgery rate was significantly lower in patients with CD receiving adalimumab every other week compared with the placebo group (0.4% vs. 3.8%, *P* = 0.01). In a previous retrospective study in Japan, infliximab was associated with improvement of the cumulative nonoperative rate in patients with CD [29]. A recent systematic review and meta-analysis revealed that the 5-year cumulative risk of surgery was 7.0% for UC and 18.0% for CD after 2000, which has significantly decreased over time [30]. Furthermore, in a Korean study, a CD diagnosis after 2003—the first year an anti-tumor necrosis factor-α antibody agent was reimbursed in Korea—compared with a CD diagnosis before 2003 was an independent predictor for intestinal resection [31]. Consistent with these previous findings, the surgery rate in CD in this study was substantially lower in patients who had an onset of disease after biologics became available in 2002 (i.e., when the first biologic, infliximab, was approved) and in 2007 (i.e., when infliximab was approved for maintenance therapy). However, it should be noted that the surgery rate was higher, and the decrease in surgery rate after the availability of biologics was more pronounced, in this study compared with the Korean study. These results may be because this study included core IBD treatment hospitals and therefore possibly included patients with more severe disease. In contrast to CD, the surgery rate in UC in this study was slightly higher in patients with onset of disease after biologics were available compared with patients with earlier disease onset, although the overall surgery rate was low and comparative data were limited to 9 years after onset. This may be explained again by the fact that patient data were collected from core IBD treatment hospitals; data from patients with more severe UC may have been concentrated in these hospitals, with an increased overall number of patients in Japan over time resulting in the higher surgery rate in patients with onset of disease after biologics were available. These results indicate that the use of biologics may reduce corticosteroid use, prevent corticosteroid-related and/or postoperative complications, and improve treatment outcomes in IBD, especially in CD. However, given that data in this study were collected retrospectively, and only in the Hokkaido region in Japan, large-scale or prospective studies may be required to further investigate the impact of biologics on surgery rates in patients with IBD in Japan.

The main strength of this study was the use of a real-world database of patients with IBD, which contains data on patients’ clinical findings, including treatment details, dosing status, treatment duration, and outcomes, for approximately 30 years. This study also included all patients with IBD diagnosed at age ≥ 10 years. The extent of the database enabled analysis of time-dependent treatment patterns in patients with IBD, including those with onset of disease before 1990, who are undergoing long-term treatment. A limitation of the study was that data were only collected from five core IBD treatment hospitals in Hokkaido, Japan; therefore, the results may not reflect clinical practice in other hospitals and may be subject to selection bias. In addition, the patients included in this study may not reflect the true IBD population in Japan and, as noted above, they may have more severe IBD. The sample size was small for some subgroups, especially patients with an earlier disease onset, because the study was initiated in 2018. The accuracy of the data before 1990 may also be limited because the database was started from 1990. Additionally, the accuracy of data on enteral nutrition in patients with CD (e.g., intake per patient) may also be limited. Lastly, patients’ medical information (e.g., previous or current treatment history) was not tracked or recorded if the patient was treated at a different hospital, and the follow-up time for some analyses was limited.

## Conclusion

In conclusion, this study provides insights into changes in IBD treatment, particularly corticosteroid use, over approximately 30 years in real-world clinical settings in Japan. In this study, corticosteroids appear to be used more appropriately, particularly since the appearance of biologics in the late 2000s. Results also suggest that the use of an initial corticosteroid dose of ≥ 30 mg/day may reduce the duration of corticosteroid treatment in CD. Furthermore, use of biologics may be effective in avoiding surgery, and thus postoperative complications, particularly in patients with CD.

## Role of the sponsor

AbbVie was involved in the study design, data collection, and analysis, interpretation of the study results, and in the drafting, critical revision, and approval of the final version of the manuscript.

### Supplementary Information

Below is the link to the electronic supplementary material.

Supplementary file1 (PDF 410 KB)

## Data Availability

The data underlying this article will be shared on reasonable request to the corresponding author.
