# Supplementary material for: Change in systemic steroid use and surgery rate in patients with inflammatory bowel disease: a Japanese real-world database analysis
Source: J Gastroenterol. 2024 Mar 16;59(5):389–401. doi: 10.1007/s00535-024-02086-y (PMC11033244; doi:10.1007/s00535-024-02086-y)

Supplementary Materials

Supplementary Table 1 Patient baseline demographics and clinical characteristics in overall population with UC, by year of onset of disease

Characteristics	1965–1990 (*n* = 37)	1991–1995 (*n* = 38)	1996–2000 (*n* = 63)	2001–2005 (*n* = 88)	2006–2010 (*n* = 181)	2011–2015 (*n* = 358)	2016–2020 (*n* = 290)	Unknown (*n* = 11)
Sex, *n* (%)								
Male	15 (40.5)	19 (50.0)	32 (50.8)	39 (44.3)	94 (51.9)	173 (48.3)	138 (47.6)	4 (36.4)
Female	22 (59.5)	19 (50.0)	31 (49.2)	49 (55.7)	87 (48.1)	185 (51.7)	152 (52.4)	4 (36.4)
Unknown	0 (0.0)	0 (0.0)	0 (0.0)	0 (0.0)	0 (0.0)	0 (0.0)	0 (0.0)	3 (27.3)
Duration of disease								
n (%)	37 (100.0)	38 (100.0)	63 (100.0)	88 (100.0)	181 (100.0)	358 (100.0)	290 (100.0)	0 (0.0)
Median (IQR), years	32.0 (30.0–36.0)	26.5 (25.0–28.0)	21.0 (20.0–22.0)	16.0 (14.5–17.0)	11.0 (9.0–12.0)	6.0 (5.0–7.0)	2.0 (1.0–3.0)	–
Age at onset of disease								
n (%)	37 (100.0)	38 (100.0)	63 (100.0)	88 (100.0)	181 (100.0)	358 (100.0)	290 (100.0)	5 (45.5)
Median (IQR), years	33.0 (17.0–39.0)	26.5 (21.0–37.0)	34.0 (24.0–44.0)	32.0 (23.0–45.5)	34.0 (23.0–43.0)	34.5 (24.0–47.0)	39.0 (25.0–52.0)	51.0 (44.0–53.0)
Height								
n (%)	22 (59.5)	24 (63.2)	31 (49.2)	52 (59.1)	119 (65.7)	302 (84.4)	265 (91.4)	5 (45.5)
Median (IQR), cm	158.5 (151.0–170.0)	165.5 (156.5–169.5)	163.0 (153.0–169.0)	162.0 (156.5–166.9)	164.0 (159.0–170.0)	163.0 (157.0–170.0)	163.0 (156.5–170.0)	157.0 (154.2–176.0)

Characteristics	1965–1990 (*n* = 37)	1991–1995 (*n* = 38)	1996–2000 (*n* = 63)	2001–2005 (*n* = 88)	2006–2010 (*n* = 181)	2011–2015 (*n* = 358)	2016–2020 (*n* = 290)	Unknown (*n* = 11)
Weight								
n (%)	21 (56.8)	24 (63.2)	28 (44.4)	52 (59.1)	116 (64.1)	298 (83.2)	263 (90.7)	5 (45.5)
Median (IQR), kg	55.0 (48.6–60.0)	57.0 (49.0–65.0)	58.5 (50.5–67.0)	58.3 (53.0–68.5)	56.0 (49.8–64.5)	56.0 (49.0–68.0)	58.7 (49.2–67.0)	58.0 (53.0–62.0)
Drinking history, *n* (%)								
Yes	7 (18.9)	11 (28.9)	13 (20.6)	21 (23.9)	53 (29.3)	118 (33.0)	114 (39.3)	2 (18.2)
No	15 (40.5)	13 (34.2)	29 (46.0)	33 (37.5)	86 (47.5)	196 (54.7)	163 (56.2)	2 (18.2)
Unknown	15 (40.5)	14 (36.8)	21 (33.3)	34 (38.6)	42 (23.2)	44 (12.3)	13 (4.5)	7 (63.6)
Smoking history, *n* (%)								
Current smoker	4 (10.8)	6 (15.8)	6 (9.5)	10 (11.4)	16 (8.8)	30 (8.4)	47 (16.2)	0 (0)
Past smoker	0 (0)	5 (13.2)	5 (7.9)	9 (10.2)	24 (13.3)	32 (8.9)	35 (12.1)	1 (9.1)
Non-smoker	19 (51.4)	14 (36.8)	31 (49.2)	37 (42.0)	98 (54.1)	261 (72.9)	193 (66.6)	2 (18.2)
Unknown	14 (37.8)	13 (34.2)	21 (33.3)	32 (36.4)	43 (23.8)	35 (9.8)	15 (5.2)	8 (72.7)
Disease type, *n* (%)								
Proctitis	5 (13.5)	10 (26.3)	8 (12.7)	14 (15.9)	40 (22.1)	106 (29.6)	67 (23.1)	3 (27.3)
Left-sided colitis	8 (21.6)	7 (18.4)	9 (14.3)	28 (31.8)	38 (21.0)	74 (20.7)	70 (24.1)	0 (0)
Total colitis	17 (45.9)	20 (52.6)	35 (55.6)	38 (43.2)	92 (50.8)	166 (46.4)	140 (48.3)	1 (9.1)
Regional colitis	0 (0.0)	0 (0.0)	2 (3.2)	1 (1.1)	1 (0.6)	0 (0.0)	0 (0.0)	0 (0.0)
Unknown	7 (18.9)	1 (2.6)	9 (14.3)	7 (8.0)	10 (5.5)	12 (3.4)	13 (4.5)	7 (63.6)

Characteristics	1965–1990 (*n* = 37)	1991–1995 (*n* = 38)	1996–2000 (*n* = 63)	2001–2005 (*n* = 88)	2006–2010 (*n* = 181)	2011–2015 (*n* = 358)	2016–2020 (*n* = 290)	Unknown (*n* = 11)
Extraintestinal complications, *n* (%)								
Yes	4 (10.8)	3 (7.9)	6 (9.5)	6 (6.8)	9 (5.0)	17 (4.7)	28 (9.7)	0 (0.0)
No	26 (70.3)	31 (81.6)	43 (68.3)	63 (71.6)	151 (83.4)	295 (82.4)	230 (79.3)	5 (45.5)
Unknown	7 (18.9)	4 (10.5)	14 (22.2)	19 (21.6)	21 (11.6)	46 (12.8)	32 (11.0)	6 (54.5)

IQR interquartile range, *UC* ulcerative colitis

Supplementary Table 2 Patient baseline demographics and clinical characteristics in overall population with cd, by year of onset of disease

Characteristics	1965–1990 (*n* = 41)	1991–1995 (*n* = 40)	1996–2000 (*n* = 43)	2001–2005 (*n* = 55)	2006–2010 (*n* = 77)	2011–2015 (*n* = 191)	2016–2020 (*n* = 130)	Unknown (*n* = 2)
Sex, *n* (%)								
Male	32 (78.0)	29 (72.5)	34 (79.1)	40 (72.7)	49 (63.6)	138 (72.3)	104 (80.0)	1 (50.0)
Female	9 (22.0)	11 (27.5)	9 (20.9)	15 (27.3)	27 (35.1)	53 (27.7)	26 (20.0)	1 (50.0)
Unknown	0 (0.0)	0 (0.0)	0 (0.0)	0 (0.0)	1 (1.3)	0 (0.0)	0 (0.0)	0 (0.0)
Duration of disease								
n (%)	41 (100.0)	40 (100.0)	43 (100.0)	55 (100.0)	77 (100.0)	191 (100.0)	130 (100.0)	0 (0.0)
Median (IQR), years	33.0 (30.0–36.0)	26.0 (25.0–27.0)	22.0 (20.0–23.0)	16.0 (15.0–18.0)	11.0 (10.0–13.0)	6.0 (4.0–7.0)	2.0 (1.0–3.0)	–
Age at onset of disease								
n (%)	41 (100.0)	40 (100.0)	43 (100.0)	55 (100.0)	77 (100.0)	191 (100.0)	130 (100.0)	1 (50.0)
Median (IQR), years	24.0 (19.0–30.0)	20.5 (16.5–25.5)	21.0 (18.0–27.0)	25.0 (19.0–32.0)	23.0 (19.0–31.0)	22.0 (18.0–30.0)	21.0 (17.0–28.0)	26.0 (26.0–26.0)
Height								
n (%)	20 (48.8)	21 (52.5)	20 (46.5)	36 (65.5)	55 (71.4)	180 (94.2)	123 (94.6)	1 (50.0)
Median (IQR), cm	161.0 (154.8–167.0)	163.0 (161.0–167.0)	169.5 (161.5–175.0)	169.5 (163.0–175.0)	166.0 (158.0–172.0)	168.0 (160.6–173.0)	169.0 (161.0–173.0)	162.0 (162.0–162.0)
Weight								

Characteristics	1965–1990 (*n* = 41)	1991–1995 (*n* = 40)	1996–2000 (*n* = 43)	2001–2005 (*n* = 55)	2006–2010 (*n* = 77)	2011–2015 (*n* = 191)	2016–2020 (*n* = 130)	Unknown (*n* = 2)
n (%)	16 (39.0)	18 (45.0)	17 (39.5)	33 (60.0)	51 (66.2)	175 (91.6)	122 (93.8)	1 (50.0)
Median (IQR), kg	45.1 (39.0–57.4)	57.5 (50.0–64.0)	63.0 (54.0–72.0)	56.0 (52.0–71.0)	56.6 (49.0–61.0)	55.0 (48.2–63.0)	54.8 (49.0–62.0)	49.3 (49.3–49.3)
Drinking history, *n* (%)								
Yes	7 (17.1)	7 (17.5)	12 (27.9)	9 (16.4)	25 (32.5)	37 (19.4)	31 (23.8)	0 (0.0)
No	18 (43.9)	21 (52.5)	19 (44.2)	26 (47.3)	36 (46.8)	133 (69.6)	90 (69.2)	1 (50.0)
Unknown	16 (39.0)	12 (30.0)	12 (27.9)	20 (36.4)	16 (20.8)	21 (11.0)	9 (6.9)	1 (50.0)
Smoking history, *n* (%)								
Current smoker	6 (14.6)	7 (17.5)	13 (30.2)	4 (7.3)	15 (19.5)	24 (12.6)	19 (14.6)	1 (50.0)
Past smoker	2 (4.9)	3 (7.5)	1 (2.3)	4 (7.3)	4 (5.2)	4 (2.1)	6 (4.6)	0 (0.0)
Non-smoker	17 (41.5)	18 (45.0)	17 (39.5)	28 (50.9)	43 (55.8)	144 (75.4)	94 (72.3)	0 (0.0)
Unknown	16 (39.0)	12 (30.0)	12 (27.9)	19 (34.5)	15 (19.5)	19 (9.9)	11 (8.5)	1 (50.0)
Disease type, *n* (%)								
Ileal	10 (24.4)	9 (22.5)	12 (27.9)	17 (30.9)	22 (28.6)	28 (14.7)	26 (20.0)	1 (50.0)
Colonic	3 (7.3)	8 (20.0)	0 (0.0)	4 (7.3)	12 (15.6)	28 (14.7)	13 (10.0)	0 (0.0)
Ileocolonic	27 (65.9)	20 (50.0)	30 (69.8)	34 (61.8)	43 (55.8)	133 (69.6)	91 (70.0)	0 (0.0)
Isolated upper disease	0 (0.0)	0 (0.0)	1 (2.3)	0 (0.0)	0 (0.0)	0 (0.0)	0 (0.0)	0 (0.0)
Unknown	1 (2.4)	3 (7.5)	0 (0.0)	0 (0.0)	0 (0.0)	2 (1.0)	0 (0.0)	1 (50.0)

Characteristics	1965–1990 (*n* = 41)	1991–1995 (*n* = 40)	1996–2000 (*n* = 43)	2001–2005 (*n* = 55)	2006–2010 (*n* = 77)	2011–2015 (*n* = 191)	2016–2020 (*n* = 130)	Unknown (*n* = 2)
Extraintestinal complications, *n* (%)								
Yes	2 (4.9)	4 (10.0)	2 (4.7)	8 (14.5)	11 (14.3)	29 (15.2)	17 (13.1)	1 (50.0)
No	32 (78.0)	29 (72.5)	35 (81.4)	39 (70.9)	55 (71.4)	153 (80.1)	105 (80.8)	0 (0.0)
Unknown	7 (17.1)	7 (17.5)	6 (14.0)	8 (14.5)	11 (14.3)	9 (4.7)	8 (6.2)	1 (50.0)
Anal lesion, *n* (%)								
Yes	15 (36.6)	19 (47.5)	14 (32.6)	19 (34.5)	32 (41.6)	93 (48.7)	66 (50.8)	1 (50.0)
No	21 (51.2)	16 (40.0)	26 (60.5)	26 (47.3)	30 (39.0)	86 (45.0)	43 (33.1)	1 (50.0)
Unknown	5 (12.2)	5 (12.5)	3 (7.0)	10 (18.2)	15 (19.5)	12 (6.3)	21 (16.2)	0 (0.0)

CD Crohn’s disease, *IQR* interquartile range

Supplementary Table 3 Patient baseline demographics and clinical characteristics in corticosteroid-treated patients with UC and CD

Characteristics	UC (*n* = 412)	CD (*n* = 124)
Sex, *n* (%)		
Male	207 (50.2)	72 (62.1)
Female	205 (49.8)	47 (37.9)
Duration of disease		
n (%)	412 (100.0)	124 (100.0)
Median (IQR), years	8.0 (4.0–14.0)	14.0 (5.0–23.5)
Age at onset of disease		
n (%)	412 (100.0)	124 (100.0)
Median (IQR), years	33.5 (22.0–48.5)	21.0 (17.0–27.5)
Height		
n (%)	311 (75.5)	81 (65.3)
Median (IQR), cm	163.0 (157.0–171.0)	165.0 (158.0–172.0)
Weight		
n (%)	302 (73.3)	77 (62.1)
Median (IQR), kg	56.0 (49.2–65.0)	53.0 (46.0–62.0)
Drinking history, *n* (%)		
Yes	118 (28.6)	21 (16.9)
No	224 (54.4)	72 (58.1)
Unknown	70 (17.0)	31 (25.0)
Smoking history, *n* (%)		
Current smoker	48 (11.7)	19 (15.3)
Past smoker	49 (11.9)	3 (2.4)
Non-smoker	247 (60.0)	71 (57.3)
Unknown	68 (16.5)	31 (25.0)
Disease type, *n* (%)		
Proctitis	43 (10.4)	–
Left-sided colitis	86 (20.9)	–
Total colitis	260 (63.1)	–
Regional colitis	2 (0.5)	–
Ileal	–	16 (12.9)
Colonic	–	18 (14.5)

Characteristics	UC (*n* = 412)	CD (*n* = 124)
Ileocolonic	–	85 (68.5)
Isolated upper disease	–	1 (0.8)
Unknown	21 (5.1)	4 (3.2)
Extraintestinal complications, *n* (%)		
Yes	39 (9.5)	21 (16.9)
No	325 (78.9)	84 (67.7)
Unknown	48 (11.7)	19 (15.3)
Anal lesions		
Yes	–	47 (37.9)
No	–	60 (48.4)
Unknown	–	17 (13.7)

CD Crohn's disease, *IQR* interquartile range, *UC* ulcerative colitis

Supplementary Table 4 Patient baseline demographics and clinical characteristics in corticosteroid-treated patients with UC, by year of onset of disease

Characteristics	1965–1990 (*n* = 20)	1991–1995 (*n* = 21)	1996–2000 (*n* = 32)	2001–2005 (*n* = 37)	2006–2010 (*n* = 84)	2011–2015 (*n* = 135)	2016–2020 (*n* = 83)
Sex, *n* (%)							
Male	9 (45.0)	10 (47.6)	16 (50.0)	13 (35.1)	40 (47.6)	75 (55.6)	44 (53.0)
Female	11 (55.0)	11 (52.4)	16 (50.0)	24 (64.9)	44 (52.4)	60 (44.4)	39 (47.0)
Duration of disease							
n (%)	20 (100.0)	21 (100.0)	32 (100.0)	37 (100.0)	84 (100.0)	135 (100.0)	83 (100.0)
Median (IQR), years	30.5 (29.0–33.5)	26.0 (24.0–27.0)	21.0 (20.0–22.5)	15.0 (14.0–17.0)	11.0 (10.0–12.5)	6.0 (4.0–7.0)	2.0 (1.0–3.0)
Age at onset of disease							
n (%)	20 (100.0)	21 (100.0)	32 (100.0)	37 (100.0)	84 (100.0)	135 (100.0)	83 (100.0)
Median (IQR), years	33.0 (21.5–39.0)	26.0 (20.0–36.0)	34.5 (26.5–45.5)	42.0 (23.0–53.0)	35.5 (23.0–49.0)	30.0 (20.0–47.0)	35.0 (22.0–53.0)
Height							
n (%)	12 (60.0)	15 (71.4)	15 (46.9)	22 (59.5)	60 (71.4)	112 (83.0)	75 (90.4)
Median (IQR), cm	156.5 (151.6–168.0)	167.0 (158.0–173.0)	160.0 (156.0–167.0)	161.5 (156.0–166.0)	163.0 (158.5–168.9)	163.3 (158.0–172.0)	165.0 (156.0–172.0)
Weight							
n (%)	12 (60.0)	15 (71.4)	13 (40.6)	22 (59.5)	58 (69.0)	110 (81.5)	72 (86.7)
Median (IQR), kg	49.8 (45.5–55.0)	57.0 (49.0–70.0)	58.0 (55.5–62.0)	56.0 (50.0–61.0)	55.5 (49.3–62.0)	55.0 (47.0–65.0)	58.9 (50.3–67.0)

Characteristics	1965–1990 (*n* = 20)	1991–1995 (*n* = 21)	1996–2000 (*n* = 32)	2001–2005 (*n* = 37)	2006–2010 (*n* = 84)	2011–2015 (*n* = 135)	2016–2020 (*n* = 83)
Drinking history, *n* (%)							
Yes	5 (25.0)	8 (38.1)	5 (15.6)	9 (24.3)	19 (22.6)	42 (31.1)	30 (36.1)
No	8 (40.0)	7 (33.3)	18 (56.3)	15 (40.5)	48 (57.1)	78 (57.8)	50 (60.2)
Unknown	7 (35.0)	6 (28.6)	9 (28.1)	13 (35.1)	17 (20.2)	15 (11.1)	3 (3.6)
Smoking history, *n* (%)							
Current smoker	3 (15.0)	5 (23.8)	2 (6.3)	4 (10.8)	6 (7.1)	16 (11.9)	12 (14.5)
Past smoker	0 (0.0)	4 (19.0)	3 (9.4)	5 (13.5)	11 (13.1)	13 (9.6)	13 (15.7)
Non-smoker	10 (50.0)	8 (38.1)	18 (56.3)	15 (40.5)	50 (59.5)	92 (68.1)	54 (65.1)
Unknown	7 (35.0)	4 (19.0)	9 (28.1)	13 (35.1)	17 (20.2)	14 (10.4)	4 (4.8)
Disease type, *n* (%)							
Proctitis	3 (15.0)	3 (14.3)	2 (6.3)	1 (2.7)	12 (14.3)	18 (13.3)	4 (4.8)
Left-sided colitis	1 (5.0)	4 (19.0)	2 (6.3)	14 (37.8)	19 (22.6)	25 (18.5)	21 (25.3)
Total colitis	12 (60.0)	13 (61.9)	22 (68.8)	19 (51.4)	52 (61.9)	89 (65.9)	53 (63.9)
Regional colitis	0 (0.0)	0 (0.0)	2 (6.3)	0 (0.0)	0 (0.0)	0 (0.0)	0 (0.0)
Unknown	4 (20.0)	1 (4.8)	4 (12.5)	3 (8.1)	1 (1.2)	3 (2.2)	5 (6.0)
Extraintestinal complications, *n* (%)							
Yes	3 (15.0)	2 (9.5)	5 (15.6)	4 (10.8)	6 (7.1)	10 (7.4)	9 (10.8)
No	14 (70.0)	18 (85.7)	24 (75.0)	25 (67.6)	71 (84.5)	110 (81.5)	63 (75.9)
Unknown	3 (15.0)	1 (4.8)	3 (9.4)	8 (21.6)	7 (8.3)	15 (11.1)	11 (13.3)

IQR interquartile range, *UC* ulcerative colitis

Supplementary Table 5 Patient baseline demographics and clinical characteristics in corticosteroid-treated patients with CD, by year of onset of disease

Characteristics	1965–1990 (*n* = 19)	1991–1995 (*n* = 15)	1996–2000 (*n* = 12)	2001–2005 (*n* = 17)	2006–2010 (*n* = 9)	2011–2015 (*n* = 39)	2016–2020 (*n* = 13)
Sex, *n* (%)							
Male	15 (78.9)	7 (46.7)	9 (75.0)	10 (58.8)	5 (55.6)	22 (56.4)	9 (69.2)
Female	4 (21.1)	8 (53.3)	3 (25.0)	7 (41.2)	4 (44.4)	17 (43.6)	4 (30.8)
Duration of disease							
n (%)	19 (100.0)	15 (100.0)	12 (100.0)	17 (100.0)	9 (100.0)	39 (100.0)	13 (100.0)
Median (IQR), years	33.0 (30.0–36.0)	27.0 (24.0–28.0)	22.0 (20.0–23.0)	15.0 (15.0–17.0)	10.0 (10.0–14.0)	5.0 (4.0–7.0)	3.0 (2.0–3.0)
Age at onset of disease							
n (%)	19 (100.0)	15 (100.0)	12 (100.0)	17 (100.0)	9 (100.0)	39 (100.0)	13 (100.0)
Median (IQR), years	24.0 (19.0–28.0)	22.0 (17.0–29.0)	20.5 (15.0–22.0)	22.0 (17.0–26.0)	21.0 (20.0–28.0)	20.0 (17.0–28.0)	21.0 (18.0–27.0)
Height							
n (%)	7 (36.8)	9 (60.0)	4 (33.3)	10 (58.8)	4 (44.4)	35 (89.7)	12 (92.3)
Median (IQR), cm	165.0 (150.0–167.0)	163.0 (160.0–165.0)	169.0 (162.5–175.5)	164.5 (161.0–171.0)	168.0 (155.5–178.5)	165.0 (156.0–173.0)	167.5 (158.0–172.0)
Weight							
n (%)	7 (36.8)	7 (46.7)	4 (33.3)	9 (52.9)	4 (44.4)	34 (87.2)	12 (92.3)
Median (IQR), kg	47.0 (37.6–59.0)	59.0 (46.0–62.0)	63.0 (57.5–69.0)	53.0 (52.0–69.0)	51.2 (50.0–58.2)	52.0 (45.0–59.0)	53.0 (46.0–62.2)

Characteristics	1965–1990 (*n* = 19)	1991–1995 (*n* = 15)	1996–2000 (*n* = 12)	2001–2005 (*n* = 17)	2006–2010 (*n* = 9)	2011–2015 (*n* = 39)	2016–2020 (*n* = 13)
Drinking history, *n* (%)							
Yes	4 (21.1)	4 (26.7)	1 (8.3)	1 (5.9)	3 (33.3)	5 (12.8)	3 (23.1)
No	5 (26.3)	9 (60.0)	7 (58.3)	10 (58.8)	3 (33.3)	30 (76.9)	8 (61.5)
Unknown	10 (52.6)	2 (13.3)	4 (33.3)	6 (35.3)	3 (33.3)	4 (10.3)	2 (15.4)
Smoking history, *n* (%)							
Current smoker	4 (21.1)	4 (26.7)	2 (16.7)	1 (5.9)	2 (22.2)	4 (10.3)	2 (15.4)
Past smoker	0 (0.0)	2 (13.3)	0 (0.0)	1 (5.9)	0 (0.0)	0 (0.0)	0 (0.0)
Non-smoker	5 (26.3)	7 (46.7)	6 (50.0)	9 (52.9)	4 (44.4)	31 (79.5)	9 (69.2)
Unknown	10 (52.6)	2 (13.3)	4 (33.3)	6 (35.3)	3 (33.3)	4 (10.3)	2 (15.4)
Disease type, *n* (%)							
Ileal	5 (26.3)	2 (13.3)	1 (8.3)	3 (17.6)	1 (11.1)	3 (7.7)	1 (7.7)
Colonic	1 (5.3)	6 (40.0)	0 (0.0)	1 (5.9)	2 (22.2)	7 (17.9)	1 (7.7)
Ileocolonic	12 (63.2)	5 (33.3)	10 (83.3)	13 (76.5)	6 (66.7)	28 (71.8)	11 (84.6)
Isolated upper disease	0 (0.0)	0 (0.0)	1 (8.3)	0 (0.0)	0 (0.0)	0 (0.0)	0 (0.0)
Unknown	1 (5.3)	2 (13.3)	0 (0.0)	0 (0.0)	0 (0.0)	1 (2.6)	0 (0.0)
Extraintestinal complications, *n* (%)							
Yes	0 (0.0)	3 (20.0)	0 (0.0)	2 (11.8)	3 (33.3)	11 (28.2)	2 (15.4)
No	15 (78.9)	10 (66.7)	10 (83.3)	11 (64.7)	4 (44.4)	25 (64.1)	9 (69.2)
Unknown	4 (21.1)	2 (13.3)	2 (16.7)	4 (23.5)	2 (22.2)	3 (7.7)	2 (15.4)

Characteristics	1965–1990 (*n* = 19)	1991–1995 (*n* = 15)	1996–2000 (*n* = 12)	2001–2005 (*n* = 17)	2006–2010 (*n* = 9)	2011–2015 (*n* = 39)	2016–2020 (*n* = 13)
Anal lesion, *n* (%)							
Yes	7 (36.8)	6 (40.0)	4 (33.3)	4 (23.5)	4 (44.4)	16 (41.0)	6 (46.2)
No	8 (42.1)	6 (40.0)	6 (50.0)	10 (58.8)	4 (44.4)	22 (56.4)	4 (30.8)
Unknown	4 (21.1)	3 (20.0)	2 (16.7)	3 (17.6)	1 (11.1)	1 (2.6)	3 (23.1)

CD Crohn's disease, *IQR* interquartile range

Supplementary Figure Kaplan-Meier curve of surgery rate in corticosteroid-treated patients with CD

Note: “Before biologics” was any time before the year of infliximab approval for CD maintenance therapy, i.e., up to and including 2007. “After biologics” was any time after the year of infliximab approval for CD maintenance therapy, i.e., starting in 2008. *CD* Crohn’s disease, *UC* ulcerative colitis.

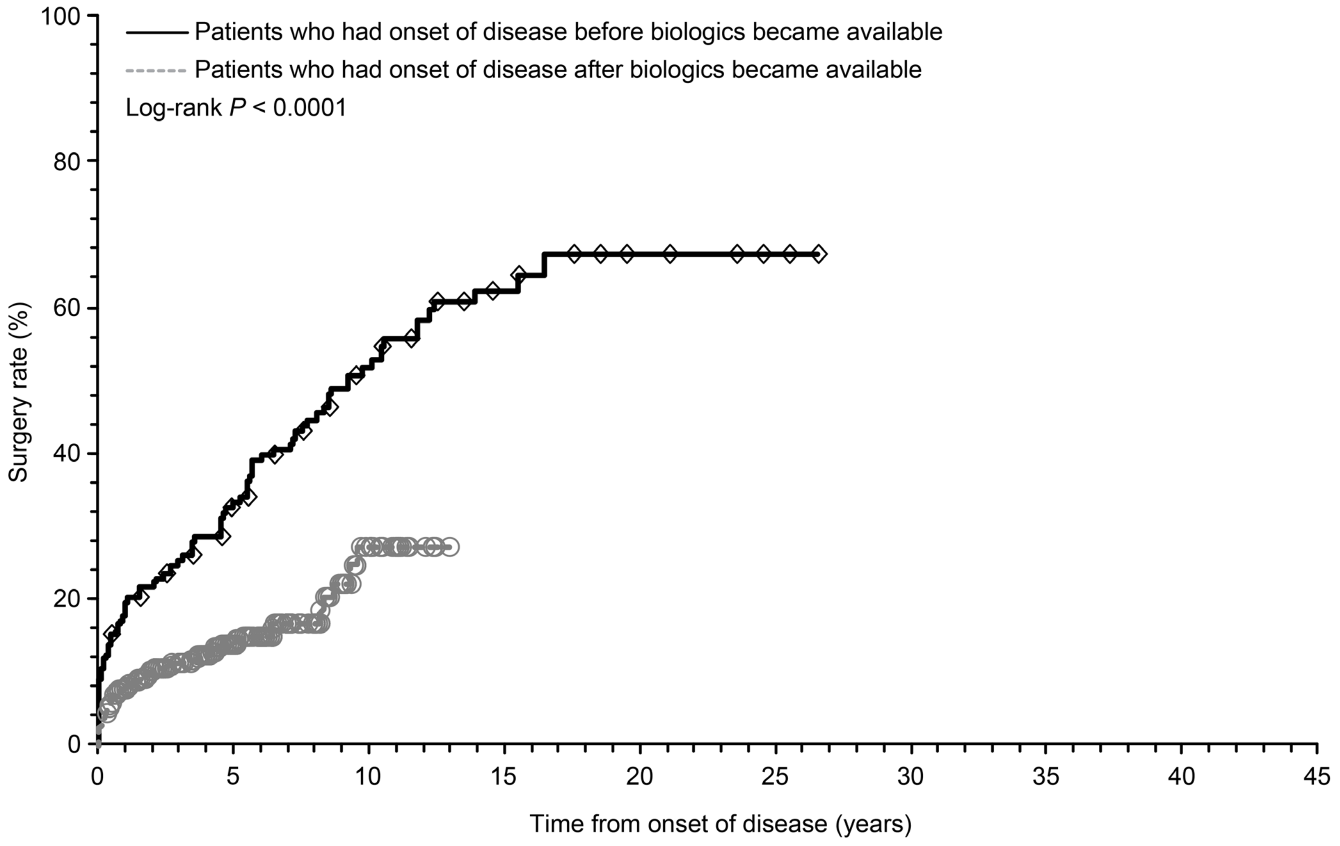

Supplement: Supplementary file 1 — Supplementary file1 (PDF 410 KB) [file 535_2024_2086_MOESM1_ESM.pdf]
